# The Engrailed-1 Gene Stimulates Brown Adipogenesis

**DOI:** 10.1155/2016/7369491

**Published:** 2016-04-11

**Authors:** Chuanhai Zhang, Yibing Weng, Fangxiong Shi, Wanzhu Jin

**Affiliations:** ^1^Laboratory of Animal Reproduction, College of Animal Science and Technology, Nanjing Agricultural University, Nanjing 210095, China; ^2^Key Laboratory of Animal Ecology and Conservation Biology, Institute of Zoology, Chinese Academy of Sciences, Beijing 100101, China; ^3^Department of Critical Care Medicine and Emergency Room, Luhe Hospital, Capital Medical University, Beijing 101149, China

## Abstract

As a thermogenic organ, brown adipose tissue (BAT) has received a great attention in treating obesity and related diseases. It has been reported that brown adipocyte was derived from engrailed-1 (EN1) positive central dermomyotome. However, functions of EN1 in brown adipogenesis are largely unknown. Here we demonstrated that EN1 overexpression increased while EN1 knockdown decreased lipid accumulation and the expressions of key adipogenic genes including PPAR*γ*2 and C/EBP*α* and mitochondrial OXPHOS as well as BAT specific marker UCP1. Taken together, our findings clearly indicate that EN1 is a positive regulator of brown adipogenesis.

## 1. Introduction

In small mammals, brown adipose tissue (BAT) is a major tissue responsible for nonshivering thermogenesis [1]. The mitochondria of BAT uncouple large amounts of fuel oxidation from ATP for generation of heat [2]. Recently, we demonstrated that transplantation of BAT could prevent obesity development and reverses preexisting obesity [3, 4]. It has been shown that increasing BAT activity by cold exposure could reduce fat mass in human adults [5–7]. These results highlight that increased amount and activity of BAT are a promising avenue to combat obesity and its related diseases such as diabetes.

Brown adipogenesis is regulated by several transcription factors such as peroxisome proliferator-activated receptor *γ* 2 (PPAR*γ*2), PR domain containing 16 (PRDM16), and CCAAT/enhancer-binding proteins (C/EBPs) [8–11]. The nuclear receptor corepressor RIP140, a ligand-dependent transcriptional repressor, also plays a crucial role in regulating the balance between energy storage and energy expenditure by repressing brown adipocyte differentiation [12–14]. Peroxisome proliferator-activated receptor gamma coactivator 1-alpha (PGC1*α*) and cell death activator A (CIDEA) are highly expressed in BAT which were known to regulate BAT differentiation [15, 16].

It has been demonstrated that myf5 positive progenitor cells are the origin of both brown adipocyte and myoblast [10]. In addition, engrailed-1 (EN1) positive central dermomyotome is also another source of brown adipocyte [17]. The EN1, a murine homologue of the* Drosophila* homeobox gene engrailed (En), is required for midbrain and cerebellum development and dorsal/ventral patterning of the limbs [18]. The mouse study also demonstrated that the expression of EN1 is critical in the correct development of the brain, limbs, and sternum [19]. However, the physiological function of EN1 during brown adipocytes differentiation has not been well studied.

To investigate the functional roles of EN1 in brown adipocyte, we take advantages of lentiviral mediated EN1 overexpression and/or knockdown technique to demonstrate here that EN1, indeed, promotes brown adipocyte differentiation by stimulating key adipogenic transcription factor, PPAR*γ*2 expression.

## 2. Materials and Methods

### 2.1. Mice

Eight-week-old male C57BL/6J donor mice were purchased from Vital River Laboratory Animal Technology Co. Ltd. Ob/Ob mice were from Nanjing Biomedical Research Institute of Nanjing University. For cold stimulation, C57BL/6J mice were placed in a cold chamber (4°C) or room temperature (RT) for up to 8 hrs with free access to food or water. For diet induced obesity (DIO) studies, 3-week-old male C57BL/6J mice from Vital River Laboratory Animal Technology were fed with either low-fat diet (LFD) or high-fat diet (HFD) for additional 8 weeks. The LFD (12450Bi) and HFD (D12492i) contain 10 kcal% fat and 60 kcal% fat, respectively (Research Diets). Mice were housed in the Office of Laboratory Animal Welfare certified animal facility with a 12-hour light/12-hour dark cycle. All animal studies were conducted with the approval of the Institutional Animal Care and Use Committee of Institute of Zoology, Chinese Academy of Sciences.

### 2.2. Lentivirus Package and Transfection

HEK293FT cells (Sci-Tech, Shanghai, China) were used in lentivirus package. The cells were maintained in Dulbecco's Modified Eagle Media (DMEM), supplemented with 10% FBS, 1x antibiotic-antimycotic solution, and 10 *μ*M nonessential amino acid. The coding region of EN1 gene was amplified from a BAT cDNA sample and cloned into pCDH-CMV-MCS-EF1-copGFP Lentivector (System Biosciences, San Francisco, CA, USA). The shEN1 sequences were as follows: F: CCGGGTTCCAGGCAAACCGCTATATCTCGAGATATAGCGGTTTGCCTGGAACTTTTTG, R: AATTCAAAAAGTTCCAGGCAAACCGCTATATCTCGAGATATAGCGGTTTGCCTGGAAC and generate the sequence-verified shRNAs in pLKO.1 (Addgene plasmid # 10878). The shuttle plasmid pCDH-CMV-MCS-EF1-copGFP or pLKO.1 or shEN1-pLKO.1 and lentivirus helper plasmid were cotransfected into HEK293FT cells to produce virus. Forty-eight hours after transient transfection, the fresh lentivirus containing supernatant was harvested for future use.

### 2.3. Flow Cytometry and Cell Sorting

The primary brown adipocytes were prepared in accordance methods with the previous publication [20]. Floating adipocytes were separated from the SVF (Stromal Vascular Fraction) by centrifugation at 300 ×g for 3 min. SVF was sequentially filtered through 70 *μ*m filters before staining with the following antibodies for 10 min on ice: Sca-1-APC (Miltenyi Biotec, 130-093-223), CD11b-FITC (Miltenyi Biotec, 130-081-201), and CD45-PE (Miltenyi Biotec, 130-091-610). Following antibody incubation, cells were washed, centrifuged at 300 ×g for 10 min, and sorted with a BD FACS Aria (BD Biosciences, CA, USA). Data analysis was performed using BD FACS Diva software.

### 2.4. Cell Culture

Cell culture related products and most other biochemical reagents were purchased from Sigma-Aldrich (St. Louis, MO, USA), unless otherwise specified. After being infected with lentivirus for 6–12 hours, the sorted primary brown fat preadipocytes were grown until 100% in 6-well plates and then treated with brown adipogenic induction cocktails (DMEM containing 10% FBS, 1 *μ*g/mL insulin, 1 *μ*M dexamethasone, 0.5 mM isobutylmethylxanthine, 0.12 mM indomethacin, and 1 nM 3,3′,5-triiodo-L-thyronine (T3)) for the first two days and the medium was replaced with differentiation medium supplemented with only insulin and T3 for additional 6 days for differentiation.

### 2.5. Real-Time-PCR

Total RNA was isolated using the RNeasy Mini Kit. cDNA was synthesized using random hexamers (Invitrogen, Carlsbad, CA, USA) for subsequent real-time quantitative PCR analysis (ABI Prism VIIA7; Applied Biosystems Inc., Foster City, CA, USA). PCR products were detected using SYBR Green and normalized by cyclophilin expression. Primers were designed using Primer Quest (Integrated DNA Technologies, Inc., Coralville, IA, USA). Primer sequences were available upon request.

### 2.6. Western Blotting

Cell and tissue lysates were prepared using RIPA buffer (150 mM sodium chloride, 1.0% Triton X-100, 0.5% sodium deoxycholate, 0.1% SDS, 50 mM Tris, and protease) and phosphatase inhibitor cocktail (Roche Diagnostics Co., CA, USA). Protein concentrations were measured with a BCA assay kit (Pierce Diagnostics Co., CA, USA). Protein was separated by 10% SDS-PAGE and transferred to PVDF membrane (Millipore, MA, USA). Membranes were blocked in 5% skim milk in TBST (0.02 M Tris base, 0.14 M NaCl, and 0.1% Tween 20, pH 7.4) followed by incubation with primary antibodies overnight at 4°C and then incubation with secondary antibodies conjugated with HRP. Primary antibodies used in the current study are EN1 (ab108598, R&D Systems, MN, USA), PPAR*γ*2 (#2443, Cell Signaling Technology, MA, USA), AP2 (A0232, ABclonal Biotech Co., MA, USA), UCP1 (ab155117, Abcam Co., MA, USA), PGC1*α* (ab54481, Abcam Co., MA, USA), OXPHOS (ab110413, Abcam Co., MA, USA), and GAPDH (#2118, Cell Signaling Technology, MA, USA). Signals were detected with Super Signal West Pico Chemiluminescent Substrate (Pierce, IL, USA).

### 2.7. Oil-Red O Staining

To detect neutral lipid, cells were stained with 0.2% (w/v) Oil-Red O (Sigma-Aldrich, St. Louis, MO, USA) for 10 min at room temperature after fixation with 4% PFA.

### 2.8. Statistical Analysis

The data are presented as means ± SD. Statistical significance was tested using ANOVA or Student's *t*-test. Statistical significance was set at *p* < 0.05.

## 3. Results

### 3.1. EN1 Is Highly Expressed in BAT Compared to WAT

**Figure 1:**
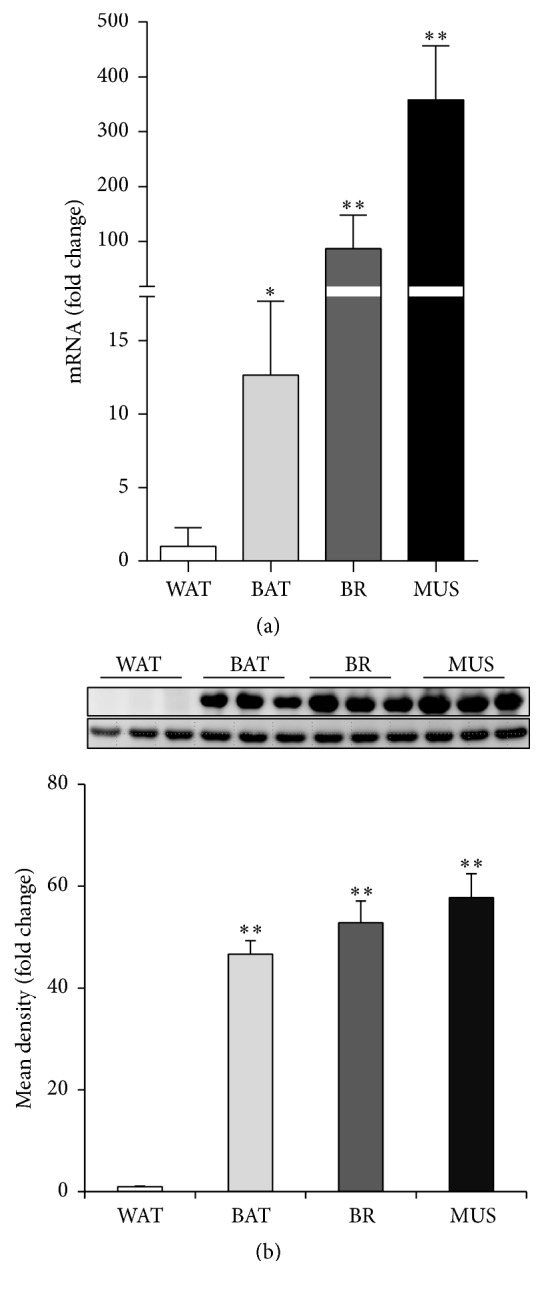
Higher expression of EN1 in BAT than in WAT. (a) The mRNA expression and (b) protein expression of EN1 in WAT, BAT, BR, and MUS were analyzed. Relative levels of EN1 protein were calculated based on densitometry analysis (bottom panel of (b)). Data are mean ± SEM. ^*∗*^ *p* < 0.05, ^*∗∗*^ *p* < 0.01, *n* = 3–5.

To investigate the expression of EN1 in related tissues, we analyzed the EN1 mRNA and protein expression in four different tissues: epididymal white adipose tissue (WAT), brown adipose tissue (BAT), brain tissue (BR), and skeletal muscles (MUS) from C57BL/6J male mice at 8 weeks of age fed with normal chow diet. The result showed that both the EN1 mRNA and protein expression were highly enriched in BAT compared with WAT (Figures 1(a)-1(b)). Surprisingly, the EN1 expression in skeletal muscle is several-hundred-fold higher than WAT.

### 3.2. Expression of EN1 in BAT at Pathophysiological Conditions

**Figure 2:**
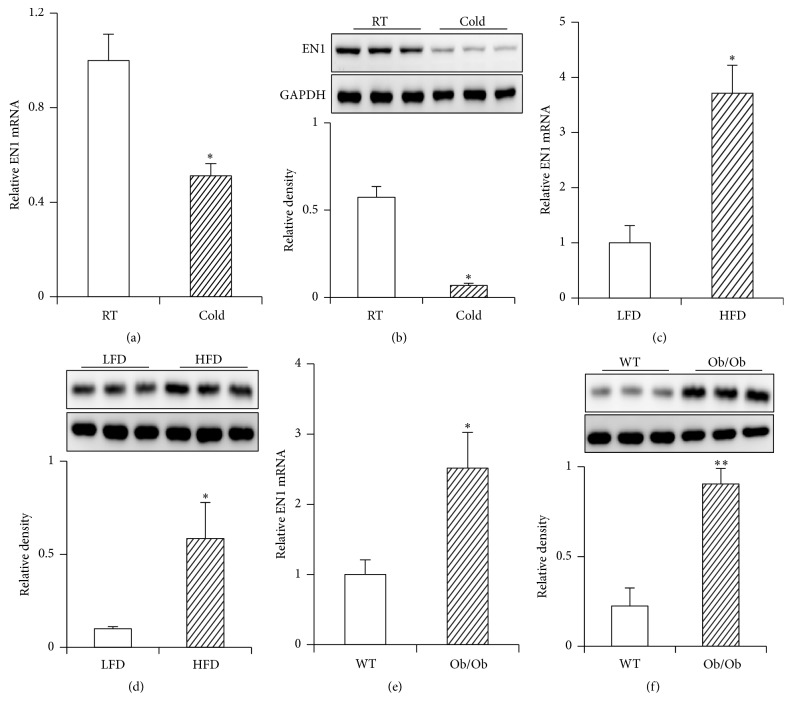
Expressions of EN1 in BAT at pathophysiological conditions. (a, c, and e) mRNA expression and (b, d, and f) protein expressions of EN1 in BAT of mice in different pathophysiological conditions: room temperature (RT) and 4°C for 8 hours (cold); 3-week-old male C57BL/6J mice fed low-fat diet (LFD) or high-fat diet (HFD) for 8 weeks; 8-week-old C57BL/6J (WT) and Ob/Ob mice were analyzed. Relative levels of EN1 protein were calculated based on densitometry analysis (bottom panel of (b), (d), and (f)). Data are mean ± SEM. ^*∗*^ *p* < 0.05, ^*∗∗*^ *p* < 0.01, *n* = 3–5.

Cold exposure is believed to be a most attractive physiological way to activate BAT [5, 21]. Moreover, previous reports have indicated dysfunction of BAT in obesity mice model [3, 4]. To determine the expression of EN1 at pathophysiological conditions, the BAT tissues were analyzed in mice with different treatment, such as room temperature (RT) versus cold exposure (Cold); low-fat diet (LFD) versus high-fat diet (HFD); wild type (WT) versus Ob/Ob mice. Interestingly, the mRNA and protein expression of EN1 were downregulated upon cold exposure (Figures 2(a)-2(b)). In contrast, they were upregulated in obesity mice compared with control mice (Figures 2(c)–2(f)). These results indicate that EN1 might influence BAT lipid contents.

### 3.3. EN1 Expression during Brown Adipogenesis

**Figure 3:**
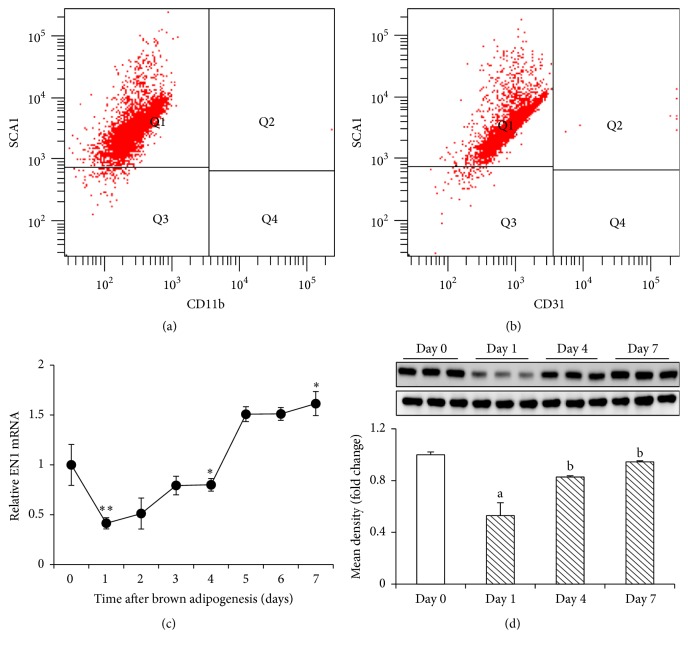
EN1 expression during brown adipogenesis. (a-b) Primary brown adipocyte (SCA1+/CD31−/CD11b−) was isolated from fetal C57BL/6J mouse BAT and (c) the mRNA and (d) protein expression of EN1 were analyzed during brown adipogenesis. Relative levels of EN1 protein were calculated based on densitometry analysis (bottom panel of (d)). Data were analyzed by unpaired Student's *t*-test in (c). ^*∗*^ *p* < 0.05, ^*∗∗*^ *p* < 0.01 versus control or analyzed by one-way ANOVA with Tukey's post hoc test; *n* = 3–5 per group.

The above results led us to hypothesize that EN1 might be involved in brown adipogenesis. To this end, primary brown adipocytes (SCA1+/CD31−/CD11b−; referred to as classical brown adipose tissue progenitor cells) were isolated from fetal C57BL/6J mouse BAT according to previous publications [22, 23] (Figures 3(a)-3(b)). In order to explore the potential role of EN1, we first investigated the mRNA and protein expression of EN1 during brown adipogenesis. Interestingly, the expression of EN1 was downregulated at day 1 and progressively increased during brown adipogenesis up to day 7 (Figures 3(c)-3(d)). These results highlight that EN1 might be involved in brown adipogenesis.

### 3.4. Overexpression of EN1 Stimulates Brown Adipogenesis

**Figure 4:**
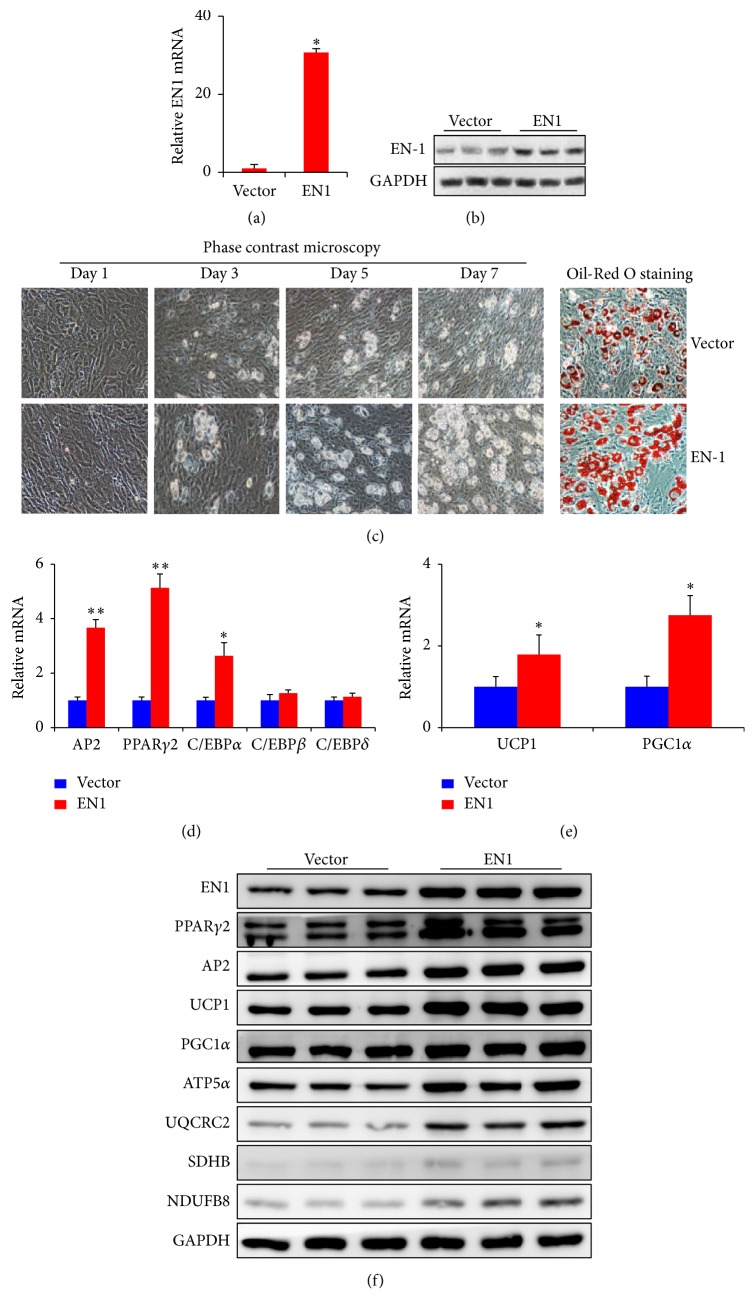
Overexpression of EN1 stimulates brown adipogenesis. Lentiviral mediated overexpression of EN1 was confirmed by (a) RT-PCR or (b) western blot analysis. Brown adipogenesis was assessed by (c) phase contrast microscopy and Oil-Red O staining. (d, e) Adipogenic and thermogenic gene expression and (f) BAT related protein expressions were analyzed during brown adipogenesis. Data are mean ± SEM. ^*∗*^ *p* < 0.05, ^*∗∗*^ *p* < 0.01, *n* = 3.

To investigate the possibility of whether EN1 is involved in brown adipogenesis, a lentivirus EN1 overexpression plasmid or empty vector was transfected into primary brown adipocyte and then underwent brown adipogenesis. EN1 expression was successfully upregulated more than 30-fold at three days after viral transduction (Figures 4(a)-4(b)). Interestingly, the lipid accumulation which was assessed by Oil-Red O staining was significantly increased by EN1 overexpression (Figure 4(c)), suggesting that EN1 stimulates brown adipogenesis. Therefore, we investigated the expression of adipogenic genes (AP2, PPAR*γ*2, and C/EBPs), brown adipocyte specific thermogenic proteins, UCP1 and PGC1*α*, and mitochondrial oxidative phosphorylation (OXPHOS) protein at the end of the brown adipogenesis. Our results showed that both mRNA and protein expression of AP2 and PPAR*γ*2 were dramatically upregulated after EN1 overexpression (Figures 4(d) and 4(f)). In addition, the mRNA expression of C/EBP*α* was also significantly increased after EN1 overexpression (Figure 4(d)). Furthermore, EN1 overexpression led to significant induction of thermogenic proteins, such as UCP1 and PGC1*α*, as well as mitochondrial OXPHOS proteins, including ATP5*α*, UQCRC2, SDHB, and NDUFB8 (Figures 4(e)-4(f)). These results indicated that EN1 promotes brown adipogenesis by upregulating the key adipogenic gene expression.

### 3.5. Knockdown of EN1 Suppresses Brown Adipogenesis

**Figure 5:**
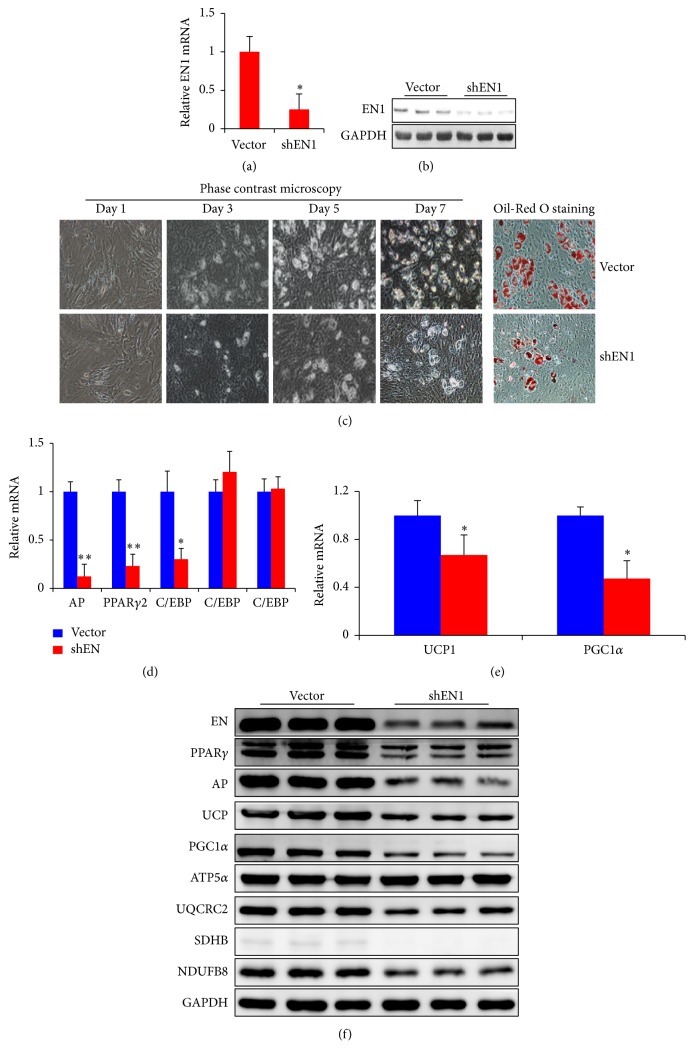
Knockdown of EN1 suppresses brown adipogenesis. Lentiviral mediated knockdown of EN1 expression was confirmed by (a) RT-PCR or (b) western blot analysis. Brown adipogenesis was assessed by (c) phase contrast microscopy and Oil-Red O staining. (d, e) Adipogenic and thermogenic gene expression and (f) BAT related protein expressions were analyzed during brown adipogenesis. Data are mean ± SEM. ^*∗*^ *p* < 0.05, ^*∗∗*^ *p* < 0.01, *n* = 3.

In order to further verify our results, knockdown of EN1, the complementary approach to overexpression, was applied to brown adipogenesis. Lentivirus encoding Sh-EN1 plasmid or empty vector was transinfected into primary brown adipocytes and underwent brown adipogenesis. We confirmed that EN1 expression was successfully downregulated about 75% at three days after viral transduction (Figures 5(a)-5(b)). As expected, lipid accumulation was significantly decreased by EN1 knockdown (Figure 5(c)). Consistently, both mRNA and protein expression of AP2 and PPAR*γ*2 were dramatically downregulated after shEN1 plasmid transfection (Figures 5(d) and 5(f)). Furthermore, the mRNA expression of C/EBP*α* was also significantly decreased by EN1 knockdown (Figure 5(d)). Moreover, EN1 knockdown led to significant reduction of thermogenic proteins, UCP1 and PGC1*α*, as well as mitochondrial OXPHOS proteins, such as ATP5*α*, UQCRC2, SDHB, and NDUFB8 (Figures 5(e)-5(f)). Taken together, the results from Figures 4-5 clearly indicated that EN1 promotes brown adipogenesis.

## 4. Discussion

In the current study, we investigated the role of EN1 in brown adipogenesis. We demonstrated that EN1 is a positive regulatory factor for brown adipogenesis. To our knowledge, this is first study showing that role of EN1 in brown adipogenesis.

Transplantation of BAT can effectively improve the whole body energy metabolism and prevent metabolic disorders, such as obesity and insulin resistance [3, 4, 7]. Increased amount and/or activity of BAT are critical approaches to combat obesity and related diseases. Generating large volume of brown adipocytes by manipulating genes including EN1 and chemicals could be one of the best approaches in the future.

EN1 is well known as one of the brown adipocyte lineage-tracing markers [17], yet little is known about its functions in brown adipogenesis. We found that expression of EN1 was higher in BAT than WAT (Figures 1(a)-1(b)). EN1 is required for midbrain and cerebellum development and patterning of the limbs [18, 19]. Consistently, we also confirmed a high expression of EN1 in the brain and muscle.

To investigate the potential role of EN1 in brown adipogenesis, we first determined the expression of EN1 during brown adipogenesis. Interestingly, expression of EN1 is downregulated at day 1 and progressively increased during brown adipogenesis upon day 7. These results prompt us to hypothesize that EN1 might be involved in brown adipocyte differentiation. Interestingly, we found that overexpression of EN1 accelerated while knockdown of EN1 suppressed the brown adipocyte differentiation (Figures 4-5). These results imply that EN1 positively regulates brown adipogenesis.

The major transcriptional factors such as PPAR*γ*2 and C/EBP*α* interacted with each other to commit adipocyte differentiation [24]. PPAR*γ*1 and PPAR*γ*2, two isoforms of PPAR*γ*, are essential transcriptional factors for both white and brown adipogenesis [25–28]. However, mutation of PPAR*γ* receptor in mice results in impairments of brown adipocyte thermogenic function and recruitment in BAT [29]. In our hands, overexpression of EN1 upregulates while knockdown of EN1 downregulates the PPAR*γ*2 and C/EBP*α* expression (Figures 4-5). These results indicate that EN1 is involved in brown adipogenesis via regulating expression of PPAR*γ*2 and C/EBP*α*. However, the exact underlying molecular regulatory mechanisms are remained to be studied.

PGC1*α*, a coactivator of PPAR*γ*2, also plays an important role in adaptive thermogenesis in BAT by regulating mitochondrial biogenesis and upregulates the expression of UCP1 [8, 30–33]. Furthermore, PRDM16 and C/EBP*β* complex synergistically enhances the activity of PGC1*α* [11, 32]. Interesting to us, the degree of EN1 expression has significant effect on BAT function as determined by mitochondrial OXPHOS protein expression and BAT specific marker gene expression including UCP1 and PGC1*α* (Figures 4-5).

In addition, the expression of EN1 is dramatically downregulated upon cold exposure, while it is increased in BAT of obesity mice. Moreover, it was significantly increased in BAT of both the high-fat diet (HFD) and Ob/Ob mice, which showed diminished functions of BAT [34, 35]. It was well known that the brown adipogenesis often associated with BAT function [36]. We therefore emphasize that EN1 might reflect the size of lipid droplets in BAT, since it is well known that HFD cause BAT hyperplasia with enlarged lipid droplet. On the other hand, cold exposure results in smaller lipid droplet size in BAT. In line with this speculation, EN1 also might be regulated by beta 3 adrenergic signaling which is important for cold induced BAT activation. Further studies are needed to clarify these hypotheses.

In conclusion, our current study demonstrated that EN1 positively regulated brown adipogenesis and BAT functions via increasing the expressions of thermogenic proteins as well as mitochondrial OXPHOS proteins.
